# Global burden of prostate cancer attributable to smoking among males in 204 countries and territories, 1990–2019

**DOI:** 10.1186/s12885-023-10552-8

**Published:** 2023-01-26

**Authors:** Hanfei Zhang, Dingping Huang, Yingfeng Zhang, Xia Wang, Jiangtao Wu, Daqing Hong

**Affiliations:** 1 School of Medicine, University of Electronic Science and Technology of China, Chengdu, China; 2 Department of Nephrology, Sichuan Provincial People’s Hospital, University of Electronic Science and Technology of China, Chengdu, China; 3 Department of Urology, Zhongshan Hospital, Xiamen University, Xiamen, China; 4 The George Institute for Global Health, University of New South Wales, Level 5, 1 King Street, Newtown, NSW 2042 Australia; 5 Department of Urology, Xuanwu Hospital, Capital Medical University, Beijing, 100053 China; 6 Renal Department and Nephrology Institute, School of Medicine, Sichuan Provincial People’s Hospital, University of Electronic Science and Technology of China, Chengdu, 610072 China

**Keywords:** Burden of disease, GBD study, Prostate cancer, Smoking, Mortality, Disability-adjusted life-years

## Abstract

**Introduction:**

Understanding the latest global spatio-temporal pattern of prostate cancer burden attributable to smoking can help guide effective global health policy. This study aims to elucidate the trends in smoking-related prostate cancer from 1990 to 2019 using Global Burden of Disease (GBD) 2019 study data.

**Methods:**

Data on prostate cancer attributable to smoking were extracted from Global Burden of Disease Study (GBD) 2019. The numbers and age-standardized rates on smoking-related prostate cancer mortality (ASMR) and disability-adjusted life years (ASDR) were analyzed by year, age, region, country, and socio-demographic index (SDI) level. Estimated annual percentage change (EAPC) was calculated to evaluate the temporal trends of ASMR and ASDR from 1990 to 2019.

**Results:**

Of all prostate cancer deaths and DALYs globally in 2019, 6% and 6.6% were attributable to smoking, which contributed to 29,298 (95% CI 12,789 to 46,609) deaths and 571,590 (95% CI 253,490 to 917,820) disability-adjusted life-years (DALYs) in 2019. The number of smoking-related deaths and DALYs showed an upward trend, increasing by half from 1990 to 2019, while ASMR and ASDR declined in five sociodemographic indexes (SDI) regions, with the fastest decline in high SDI regions. For geographical regions, Western Europe and East Asia were the high-risk areas of prostate cancer deaths and DALYs attributable to smoking, among which China and the United States were the countries with the heaviest burden. The ASMR has decreased in all age groups, with the fastest decrease occurring in 75–79 years old. The ASMR or ASDR tended to increase in countries with the lowest SDI, but declined in countries with the highest SDI. The EAPC in ASMR or ASDR was highly negatively correlated with Human Development Index (HDI) in 2019, with coefficients 0.46.

**Conclusion:**

The number of smoking-related prostate cancer deaths and DALYs continued to increase globally, whereas its ASMR and ASDR have been decreasing. This substantial progress is particularly significant in developed regions and vary across geographic regions. Medical strategies to prevent and reduce the burden should be adjusted and implemented based on country-specific disease prevalence.

**Supplementary Information:**

The online version contains supplementary material available at 10.1186/s12885-023-10552-8.

## Introduction

Worldwide, prostate cancer (PCa) is the second most commonly diagnosed malignancy and ranked fifth among all cancer mortalities in males [1]. It is estimated that prostate cancer incidence increased by 42% from 2007 to 2017, and prostate cancer ranked first among all cancer-related deaths among men in 56 countries [2]. In 2019, the burden of prostate cancer accounted for more than 8.64 million DALYs and 0.49 million deaths for all ages [3].

The tobacco epidemic is one of the biggest public health threats the world has ever faced. Although the prevalence of smoking declined steadily between 1990 and 2019, it is noteworthy that the total number of smokers has significantly increased due to population growth [4]. Concurrently, smoking contributed substantially to the risk attributable burden for all ages, ranked second in ages 25–74 years and third in ages 75 years and older [5, 6], and was the leading risk factor for death among males [4].

One latest study indicated an inconclusive relationship between smoking exposure and the risk of prostate cancer [7]. This result was the most conservative interpretation of all evidence after accounting for between-study heterogeneity, in contrast with previous work reporting that smoking is associated with an increased risk of aggressive prostate cancer and prostate cancer-specific mortality [8–10]. Of relevance, previous studies have shown that nonbiological and biological are 2 major broad classes of mechanisms linking smoking and death from prostate cancer, and it was increasingly clear with larger sample sizes [11]. For example, a meta-analysis showed that smokers were 24% more likely than nonsmokers to die from prostate cancer [10]. Thus, while at first glance the associations between smoking and prostate cancer may be null, the associations cannot be ignored, because of the high incidence of prostate cancer and the severity of the disease.

The epidemiological pattern of prostate cancer burden attributable to smoking at the global, regional, and national levels is still unknown. The Global Burden of Disease (GBD) 2019 study has collected systematic and updated data of 369 diseases and injuries and 87 related risk factors from over 204 countries and territories [3, 6]. However, among the many risk factors for prostate cancer, only data on smoking have been included. In this study, we use the latest GBD 2019 dataset to estimate the spatio-temporal trend of prostate cancer burden due to smoking and provide stakeholders with comprehensive information to better make strategies and implement the policies. To our knowledge, this is the first study to reveal the heavy prostate cancer burden due to smoking and its distribution on a global scale.

## Materials and methods

### Data sources

Annual number of prostate cancer-related deaths, DALYs, age-standardized mortality rate (ASMR), and age-standardized DALY rate (ASDR) attributable to smoking from 1990 to 2019, by year, age, region, and country were extracted from the Global Health Data Exchange (GHDx) query tool [12]. Data from a total of 204 countries and territories were available. These countries and territories were then divided into 5 levels (low, low-middle, middle, high-middle, and high) in terms of the socio-demographic indices (SDI). Besides, the world is separated into 21 regions according to epidemiological similarities and geographical proximity. Because only one in 10,000 men under age 40 will be diagnosed, we extracted 12 age categories by five-year age groups within the ages of 40–94, and ≥ 95 years old to investigate the age patterns of mortality and DALYs. Data on patients in 4 age groups, 15–74 years and ≥ 75 years, were also included, as previous literature mentioned patients with early-onset prostate cancer continued to increase over the years and that these patients with a greater genetic component were inclined to die from the cancer. We wonder the distribution of mortality and DALYs across age groups in patients exposed to smoking, and whether they would be increased in earlier age groups as predicted. We also collected human development index (HDI) data at the national level from the World Bank.

### Definitions

In GBD 2019, prostate cancer was defined by the International Classification of Diseases (ICD) code 9^th^ and 10^th^ Revision (185–185.9, V10.46, V16.42, V76.44, and C61-C61.9, Z12.5, Z80.42, Z85.46, respectively [13]. Exposure to smoking is defined as the prevalence of current use of any smoked tobacco product and prevalence of former use of any smoked tobacco product. Among current smokers, it indicates cigarette equivalents smoked per smoker per day and cumulative pack-years of exposure, while among former smokers, the distribution of the number of years since cessation is estimated [5]. 

The socio-demographic index (SDI) estimated by GBD researchers is expressed on a scale of 0 to 1, and estimated based on lag distributed income (LDI) per capita, mean education for those ages 15 and older (EDU15 +), and total fertility rate under the age of 25 (TFU25) [3, 6, 14]. The DALY is a summary measure that quantifies the overall disease burden. It represents the sum of years of life lost due to premature death and years lived with disability [5, 14, 15]. One DALY can be regarded as the loss of 1 year in full health [2]. GBD 2019 modeling strategies for estimating DALYs have been described in detail elsewhere [14, 15].

### Statistical analyses

We computed the number of deaths, DALYs, ASMR, and ASDR with 95% confidence intervals (CIs) (generated using the 25th and 97.5th values of the ordered 1,000 estimates) to quantify the burden of prostate cancer attributable to smoking [16]. Age-standardized rates were calculated by standardization to the global age structure from GBD and the attributable proportions of ASMR and ASDR due to smoking were measured using population attributable fractions, which represent the ASMR and ASDR that could have been avoided if the exposure to smoking was reduced to an alternative ideal exposure scenario. Population attributable fractions were estimated using the GBD 2019 comparative risk assessment approach [5]. The process of age-standardization of rates is a classic epidemiological method that removes the confounding effect of differences in age structure between the populations being compared, and the formula is as follows:

$$\mathrm{ASR}=\frac{\sum_{\mathrm{i}=1}^{\mathrm{A}}{\mathrm{a}}_{\mathrm{i}}{\mathrm{w}}_{\mathrm{i}}}{\sum_{\mathrm{i}=1}^{\mathrm{A}}{\mathrm{w}}_{\mathrm{i}}}\times 100 000$$

The ASR (per 100,000 population) is equal to the sum of the product of the specific age ratio (a_i_, where i denotes the i^th^ age class) and the number of persons (or weight) (w_i_) in the same age subgroup i of the chosen reference standard population, then dividing the sum of standard population weights.

An estimated annual percentage change (EAPC) was calculated to quantify the secular trends of ASR from 1990 to 2019 through the following formula: ln(y) = α + βx + ε, where y = ln(ASR), x = calendar year, and ε is the error term. EAPC could be calculated as 100 × (exp (β)-1), and its 95% confidence interval (CI) was obtained from the linear regression model. The ASR was considered to be in an upward trend if the EAPC estimation and its lower 95% CI were both > 0. Conversely, the ASR was in a downward trend if the EAPC estimation and its upper 95% CI were both < 0. Otherwise, the ASR was considered to be stable over time. In addition, to explore the influential factors for the EAPC, we assessed the association between EAPCs and ASRs (1990), and HDI (2019) at the national level using the pearson test. At last, a hierarchy cluster analysis was conducted to cluster the countries and territories into 5 categories (a: remained stable; b: minor increase; c: significant increase; d: significant decrease; e: minor decrease) according to their temporal trends in ASMR and ASDR. In addition, we first used the decomposition methodology of Das Gupta [17] to decompose global prostate cancer death and DALYs due to smoking by population age structure, population growth, and epidemiologic changes. All statistics and visualization were performed using R program (Version 4.1.2).

## Results

### Global trends of prostate cancer attributable to smoking among males

**Fig. 1:**
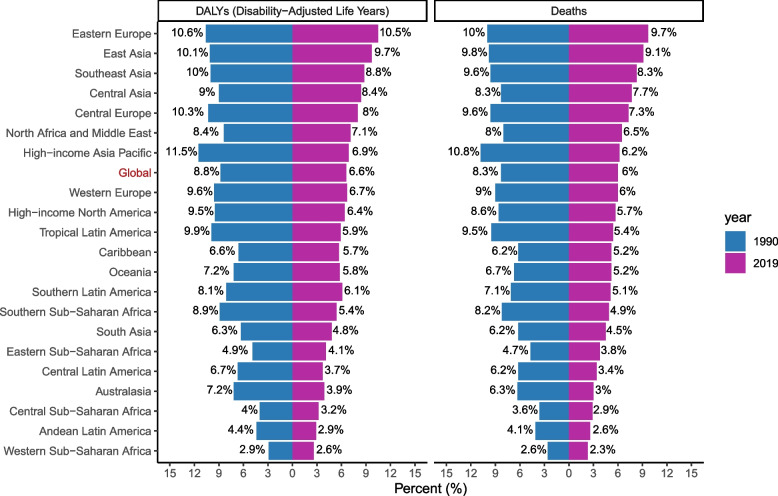
The proportion of prostate cancer deaths and DALYs attributable to smoking globally and in 21 GBD regions in 1990 and 2019. Footnote: DALYs, disability-adjusted life-years; GBD, Global Burden of Disease Study

**Fig. 2:**
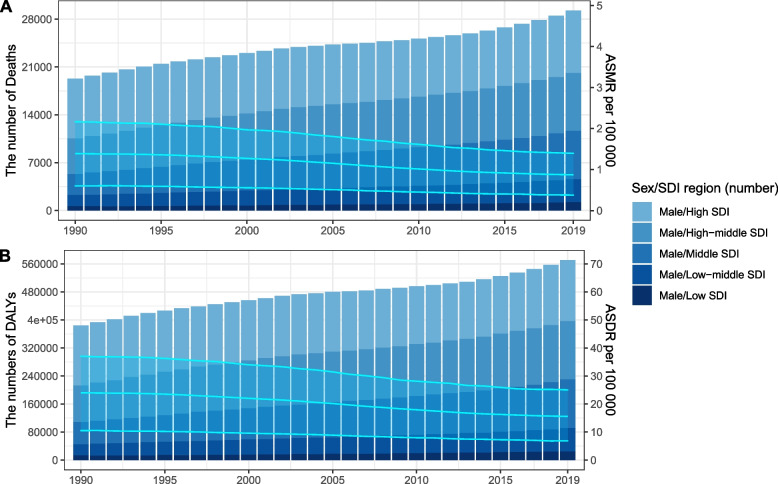
Number and rate of prostate cancer deaths (**A**) and DALYs (**B**) attributable to smoking among males from 1990 to 2019 by SDI level. Footnote: The bars represent the number of prostate cancer deaths (**A**) and DALYs (**B**) attributable to smoking colored by SDI level. The line represents the mean ASMR (**A**) and ASDR (**B**) [per 100,000] attributable to smoking at the Global level. The shaded area represents the 95% UI for the mean rate. ASMR, age-standardized mortality rate; DALYs, disability-adjusted life-years; ASDR, age-standardized DALY rate; UI, uncertainty interval; SDI, socio-demographic index

Of all prostate cancer deaths and DALYs globally in 2019, 6% and 6.6% were attributable to smoking (Fig. 1), respectively; resulting in 29,298 (12,789 to 46,609) deaths and 571,590 (253,490 to 917,820) DALYs due to prostate cancer attributable to smoking among males in 2019. The number of smoking-related prostate cancer deaths and DALYs showed an upward trend from 1990 to 2019, an increase of approximately 50% compared to those of 1990. However, the age-standardized mortality rates were 0.87 per 100,000 people in 2019 and demonstrated a downtrend with an EAPC of -1.83 (95% CI -1.94,-1.72) between 1990 and 2019. By contrast, the age-standardized DALYs rates were 15.58 per 100,000 people and demonstrated a stable trend with the EAPC of -0.8 (95% CI -2.35, 0.78) (Table 1; Fig. 2). Among the 4 age subgroups, the distribution of deaths and DALYs across age groups remained stable over time (Additional file 1).

**Table 1:** Global burden of prostate cancer attributable to smoking in 1990 and 2019, and the temporal trends from 1990 to 2019

Characteristics	**1990**				**2019**				**EAPC(1990–2019)**	
	Deaths cases	ASMR per 100 000	DALYs	ASDR per 100 000	Deaths cases	ASMR per 100 000	DALYs	ASDR per 100 000	ASMR	ASDR
	No. × 10^2^ (95% UI)	No. (95% UI)	No. × 10^3^ (95% UI)	No. (95% UI)	No. × 10^2^ (95% UI)	No. (95% UI)	No. × 10^3^ (95% UI)	No. (95% UI)	No. (95% UI)	No. (95% UI)
Global	193.16(85.06,297.38)	1.39(0.6,2.16)	384.71(169.58,592.72)	24.01(10.5,37.05)	292.98(127.89,466.09)	0.87(0.38,1.39)	571.59(253.49,917.82)	15.58(6.85,25.04)	-1.83(-1.94,-1.72)	-0.8(-2.35,0.78)
High SDI	87.63(37.88,141.14)	2.15(0.93,3.45)	170.79(74.36,275.2)	39.17(16.99,63.03)	91.62(39.64,154.2)	1.02(0.44,1.72)	174.05(75.73,289.48)	19.74(8.59,32.84)	-2.85(-2.95,-2.76)	-2.17(-4.06,-0.25)
High-middle SDI	51.86(23.97,81.07)	1.41(0.64,2.21)	105.23(49.43,163.9)	24.97(11.57,39.41)	84.42(38.86,133.22)	1.02(0.46,1.64)	167.1(77.08,263.82)	18.56(8.55,29.32)	-1.35(-1.55,-1.15)	-1.59(-3.39,0.25)
Middle SDI	31.01(13.31,48.32)	0.89(0.37,1.42)	62.61(27.54,97.2)	15.01(6.38,23.56)	70.59(29.98,113.45)	0.74(0.3,1.2)	138.82(61,219.62)	12.74(5.5,20.11)	-0.88(-1.02,-0.74)	0.52(-1.42,2.49)
Low-middle SDI	16.44(6.16,26.69)	0.77(0.28,1.27)	32.87(12.49,52.89)	12.97(4.86,21.11)	34.13(12.47,56.82)	0.65(0.23,1.09)	65.99(25.23,109.87)	11.13(4.15,18.43)	-0.78(-0.91,-0.65)	-1.21(-3.08,0.69)
Low SDI	6.06(1.83,10.9)	0.66(0.2,1.2)	12.91(3.95,23.23)	12.09(3.65,21.85)	11.93(3.48,21.04)	0.6(0.17,1.07)	25.08(7.54,44.19)	11.06(3.28,19.55)	-0.31(-0.38,-0.24)	-0.39(-1.85,1.09)
East Asia	20.79(9.74,32.37)	0.71(0.31,1.1)	42.67(20.32,66.65)	11.92(5.56,18.37)	51.86(24.05,83.38)	0.65(0.3,1.04)	101.81(47.6,163.84)	10.95(5.13,17.5)	-0.77(-0.95,-0.59)	-2.08(-3.73,-0.4)
Central Europe	9.15(4.19,14.37)	1.62(0.73,2.55)	18.36(8.54,28.71)	29.9(13.83,46.84)	13.76(6.34,22.43)	1.52(0.7,2.48)	26.51(12.35,42.32)	28.11(12.99,44.98)	-0.26(-0.45,-0.07)	0.92(-0.64,2.5)
Tropical Latin America	7.9(3.42,13.68)	2.33(0.99,4.05)	15.74(6.92,27.04)	41.12(17.78,70.94)	12.91(5.28,22.97)	1.33(0.53,2.37)	24.82(10.34,43.8)	23.88(9.81,42.47)	-2.13(-2.47,-1.79)	-1.8(-3.9,0.34)
Southeast Asia	8.72(3.6,13.95)	1(0.41,1.6)	17.56(7.35,28.12)	17.27(7.17,27.67)	22.72(8.52,38.49)	1.07(0.38,1.84)	45(17.54,74.76)	18.53(7.03,31.07)	0.26(0.19,0.32)	-1.36(-3.32,0.64)
Australasia	1.71(0.78,2.75)	1.7(0.77,2.76)	3.63(1.66,5.81)	33.94(15.51,54.55)	1.65(0.68,2.92)	0.69(0.28,1.22)	3.66(1.55,6.45)	15.68(6.64,27.63)	-3.53(-3.66,-3.41)	0.16(-1.63,1.98)
High-income Asia Pacific	7.05(3.3,10.93)	0.99(0.46,1.53)	13.48(6.36,20.95)	16.87(7.83,26.07)	11.99(5.39,19.32)	0.54(0.24,0.87)	20.62(9.25,33.07)	9.75(4.35,15.74)	-2.09(-2.26,-1.93)	-1.68(-3.43,0.11)
Oceania	0.12(0.04,0.21)	1.14(0.37,1.99)	0.27(0.09,0.47)	20.63(6.87,35.96)	0.26(0.08,0.48)	1.04(0.3,1.95)	0.59(0.18,1.08)	19.07(5.76,35.05)	-0.02(-0.13,0.1)	-0.97(-2.8,0.9)
North Africa and Middle East	5.32(2.25,8.46)	0.83(0.35,1.32)	10.62(4.59,16.9)	14.24(6.03,22.6)	12.51(5.61,19.97)	0.73(0.32,1.17)	24.62(11.06,39.14)	12.81(5.74,20.46)	-0.44(-0.64,-0.24)	-1.31(-3.37,0.8)
Eastern Sub-Saharan Africa	2.79(0.75,5.16)	0.94(0.24,1.76)	6.04(1.65,11.03)	17.73(4.78,32.78)	5.24(1.28,9.48)	0.86(0.2,1.55)	11.46(2.87,20.6)	16.58(4.06,30)	-0.41(-0.49,-0.33)	-2.51(-5.1,0.15)
Eastern Europe	10.49(4.99,17.72)	1.26(0.58,2.18)	24.09(11.73,40.47)	25.46(12.2,43.08)	19.82(9.29,30.84)	1.62(0.76,2.53)	44.98(21.21,69.52)	34.14(16.12,52.56)	1.02(0.92,1.13)	0.07(-1.3,1.47)
Western Europe	56.74(25.39,91.23)	2.51(1.12,4.03)	102.52(45.88,163.32)	42.72(19.14,68.07)	57.58(24.7,95.79)	1.31(0.56,2.17)	100.72(43.05,169.81)	23.86(10.26,40.11)	-2.63(-2.79,-2.47)	-0.82(-2.53,0.92)
High-income North America	34.8(14.21,59.78)	2.38(0.98,4.1)	74.17(29.81,127.36)	48.84(19.72,83.83)	31.25(13.09,56.14)	1.08(0.46,1.94)	65.93(26.96,117.17)	22.51(9.21,40.06)	-3.11(-3.25,-2.97)	-2.42(-4.46,-0.35)
Central Sub-Saharan Africa	0.64(0.14,1.24)	0.81(0.16,1.6)	1.42(0.33,2.72)	15.09(3.25,29.19)	1.18(0.29,2.27)	0.72(0.17,1.46)	2.62(0.66,5.02)	13.23(3.21,25.87)	-0.53(-0.63,-0.43)	-1.13(-3.01,0.79)
Western Sub-Saharan Africa	2.53(0.73,4.62)	0.72(0.2,1.33)	5.38(1.6,9.92)	13.36(3.87,24.42)	6.15(1.94,11.6)	0.87(0.27,1.65)	12.71(4.07,23.64)	16.09(5.09,30.19)	0.91(0.82,1)	-1.17(-3.67,1.39)
Southern Sub-Saharan Africa	2.69(1.08,4.41)	2.87(1.16,4.71)	5.49(2.27,8.97)	51.23(20.89,84.07)	3.6(1.3,6.08)	1.93(0.69,3.29)	7.69(2.8,12.98)	35.83(12.99,60.49)	-1.55(-1.86,-1.25)	-0.22(-2.13,1.73)
South Asia	9.64(2.8,17.13)	0.49(0.14,0.88)	19.39(5.78,34.33)	8.12(2.38,14.41)	19.22(5.68,34.77)	0.35(0.1,0.64)	36.59(11.22,65.27)	5.88(1.77,10.61)	-1.37(-1.48,-1.27)	-0.7(-2.76,1.39)
Central Asia	1.19(0.56,1.81)	0.75(0.35,1.15)	2.72(1.3,4.16)	15.34(7.3,23.38)	1.99(0.95,3.08)	0.86(0.41,1.35)	4.5(2.12,6.89)	16.15(7.66,25.02)	0.93(0.73,1.12)	-1.53(-3.34,0.32)
Central Latin America	4.07(1.36,6.99)	1.21(0.4,2.09)	7.93(2.77,13.66)	21.8(7.54,37.6)	7.33(1.91,13.61)	0.73(0.19,1.37)	14.18(3.94,26.16)	13.64(3.76,25.27)	-1.91(-2.03,-1.8)	-0.88(-2.93,1.21)
Andean Latin America	0.79(0.25,1.33)	0.94(0.29,1.59)	1.45(0.48,2.46)	16.23(5.24,27.25)	1.65(0.39,3.23)	0.66(0.16,1.3)	2.95(0.75,5.76)	11.48(2.9,22.42)	-1.1(-1.21,-0.98)	-0.31(-2.37,1.8)
Caribbean	2.48(1.06,4.03)	2.16(0.91,3.53)	4.61(1.98,7.44)	37.97(16.26,61.32)	5.14(2.09,8.46)	2.21(0.9,3.65)	9.56(3.96,15.74)	40.12(16.64,66.2)	0.09(-0.11,0.29)	0.64(-1.51,2.84)
Southern Latin America	3.55(1.54,6.02)	1.86(0.79,3.19)	7.18(3.18,12.09)	35.25(15.59,59.39)	5.17(2.09,8.88)	1.44(0.57,2.48)	10.08(4.18,17.18)	27.26(11.27,46.53)	-0.94(-1.15,-0.74)	-1.2(-3.26,0.9)

*No* Number, *ASMR* Age-standardized mortality rate, *UI* Uncertainty interval, *DALYs* Disability-adjusted life-years, *ASDR* Age-standardized DALY rate, *EAPC* Estimated annual percentage change, *CI* Confidential interval, *SDI* Sociodemographic index

### Regional trends of prostate cancer attributable to smoking among males

For SDI regions, high SDI region had the highest number of smoking-related prostate cancer deaths (9 thousand) and DALYs (174 thousand) in 2019, both accounting for over 30% worldwide. Additionally, high SDI region also carried the highest ASMR and ASDR. It was worth noting that the ASMR decreased across the 5 SDI regions from 1990 to 2019, among which high-middle and high SDI regions had a faster decrease compared to low and low-middle SDI regions. By contrast, the ASDR remain stable in low, low-middle, middle and high-middle SDI regions, but high SDI region had a rapid decrease (EAPC -2.17, 95% CI: -4.06, -0.25) (Table 1).

For geographical regions, the heaviest burden had occurred in Western Europe and East Asia for over 30 years, accounting for almost half deaths in the world. However, the highest ASMR and ASDR occurred in Caribbean. As for ASMR, the most significant decrease was detected in Australasia and High-income North America from 1990 to 2019, with EAPCs all over 3, whereas Eastern Europe had the largest increase. Concurrently, high-income North America and East Asia had the fastest decrease in ASDR, with EAPC -2.42 (95% CI: -4.46,-0.35) and -2^.^08 (95% CI: -3.73,-0.4), respectively (Table 1).

In 1990, there was an approximately fourfold difference between the regions with the highest and lowest percentage of prostate cancer deaths and DALYs attributable to smoking, with the highest percentage in Eastern and Central Europe, East and Southeast Asia, and High − income Asia Pacific and the lowest percentage in Andean Latin America, Central Sub − Saharan Africa, and Western Sub − Saharan Africa. The proportion attributable to smoking in 2019 had a similar regional distribution, except that the percentage in Central Europe and High − income Asia Pacific didn’t rank among the highest. Besides, it was worth noting that in all GBD regions, the contribution of smoking to the total number of deaths and DALYs due to prostate cancer decreased between 1990 and 2019 (Fig. 1).

### Countries and territories trends of prostate cancer among males

**Fig. 3:**
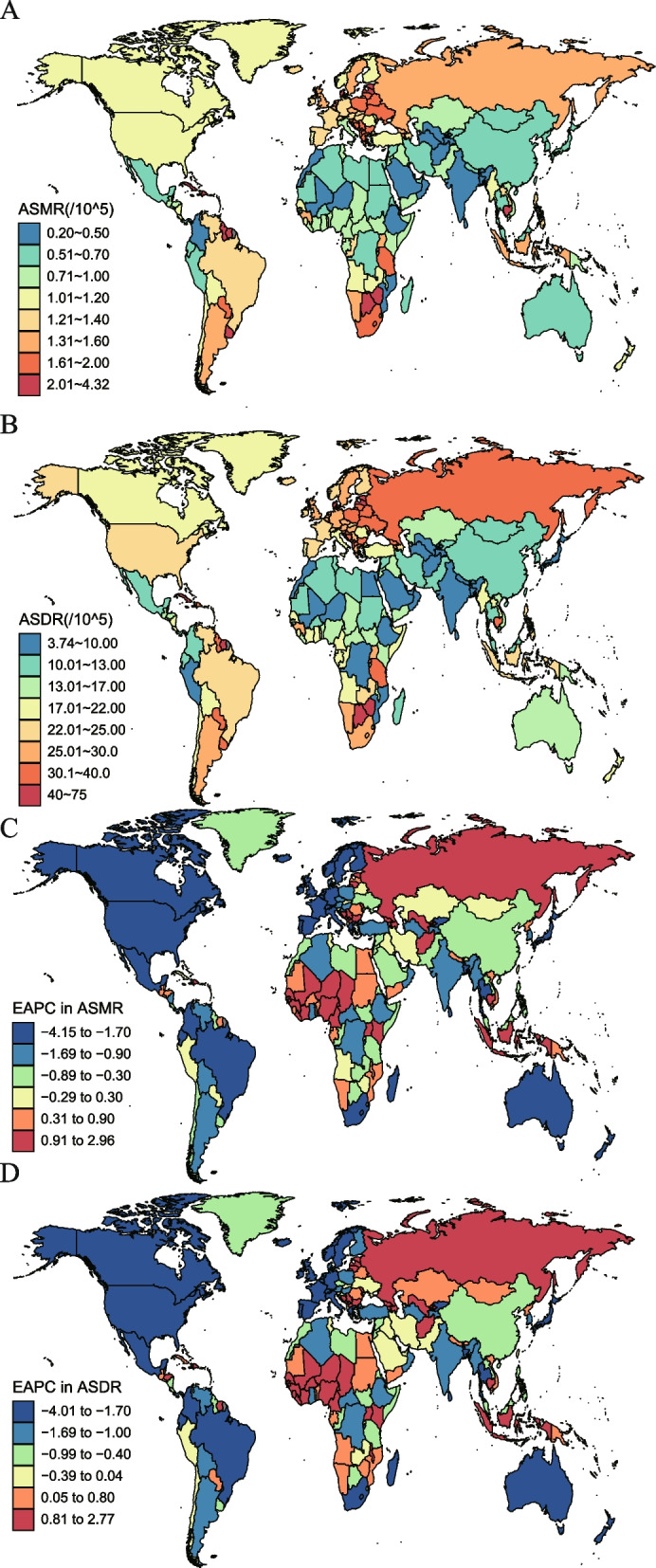
The spatial distribution of prostate cancer ASMR (**A**) and ASDR (**B**) attributable to smoking in 2019, and the EAPC in prostate cancer ASMR (**C**) and ASDR (**D**) attributable to smoking. Footnote: ASMR, age-standardized mortality rate; ASDR, age-standardized DALY rate; EAPC, estimated annual percentage change

At the country level, China ranked first in the number of prostate cancer deaths and DALYs attributable to smoking in 2019, followed by the U.S. (Additional file 2: Table S1-S2). Seychelles, Dominica, and Zimbabwe were the top three in ASMR and ASDR in 2019 (Fig. 3A and B; Additional file 2: Table S3-S4). However, the fastest increase in ASMR and ASDR occurred in Niger, with EAPCs 2.96 (95% CI: 2.78, 3.13) and 2.77 (95% CI: 2.61, 2.94) in ASMR and ASDR, respectively, and the most rapid decreased in ASMR and ASDR occurred in Canada, with EAPCs -4.15 (95% CI: -4.38, -3.92) and -4.01 (95% CI: -4.27, -3.75) in ASMR and ASDR (Fig. 3C and D; Additional file 2: Table S5-S6). Percentage change across countries of the fraction of all prostate cancer deaths and DALYs that are attributable to smoking was shown in Additional files 3 and 4.

According to the results derived from cluster analysis, 79 countries (or territories) were categorized into ‘‘remained stable” group, including China, Ukraine, Iraq, and Cuba. Thirty countries (or territories) were categorized into ‘‘minor increase” group, including Afghanistan, Egypt, and Vietnam. Sixteen countries (or territories) were categorized into ‘‘significant increase” group, including Russia and Ivory Coast. 22 countries (or territories) were categorized into ‘‘significant decrease” group, including the U.S., the UK, France, and Canada. The remaining 47 countries (or territories) were categorized into ‘‘minor decrease” group, including India, Turkey, Brazil, and Japan (Additional file 5).

### Global prostate cancer burden attributable to smoking by age

**Fig. 4:**
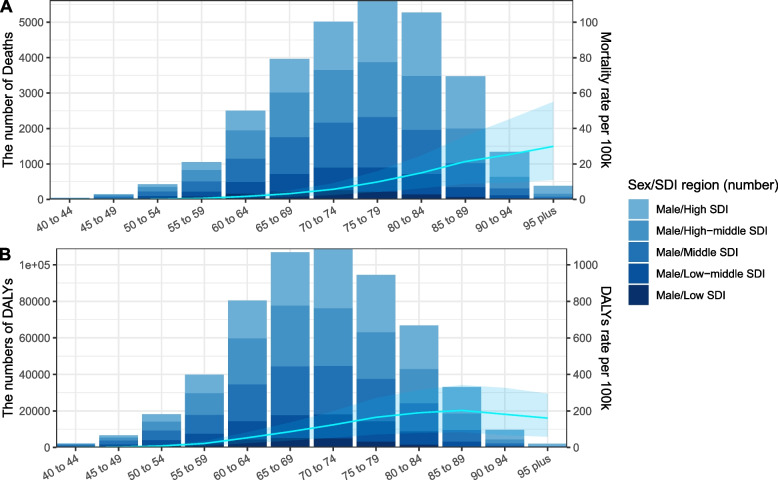
Number and rate of prostate cancer deaths (**A**) and DALYs (**B**) attributable to smoking among males by age group and SDI level in 2019. Footnote: The bars represent the number of prostate cancer deaths (**A**) and DALYs (**B**) attributable to smoking colored by SDI level. The line represents the mean ASMR (**A**) and ASDR (**B**) [per 100,000] attributable to smoking at the Global level. The shaded area represents the 95% UI for the mean rate. DALYs, disability-adjusted life-years; ASDR, age-standardized DALY rate; UI, uncertainty interval; SDI, socio-demographic index

In 2019, the number of prostate cancer deaths attributable to smoking first increased and then decrease with age. Most deaths occurred in ages 70–84 years old, with the peak at the age group 75–79, and more age-specific deaths occurred in high and high-middle SDI regions compared to that in low and low-middle regions (Fig. 4A). Correspondingly, the age-specific mortality rate kept increasing from 40 to 95 years old. A similar pattern to that of deaths, with most DALYs occurring for 65–74 year olds with a peak at 70–74. Correspondingly, age-standardized DALY rates gradually decreased after reaching their highest value in the age range of 85–89 years old (Fig. 4B).

**Fig. 5:**
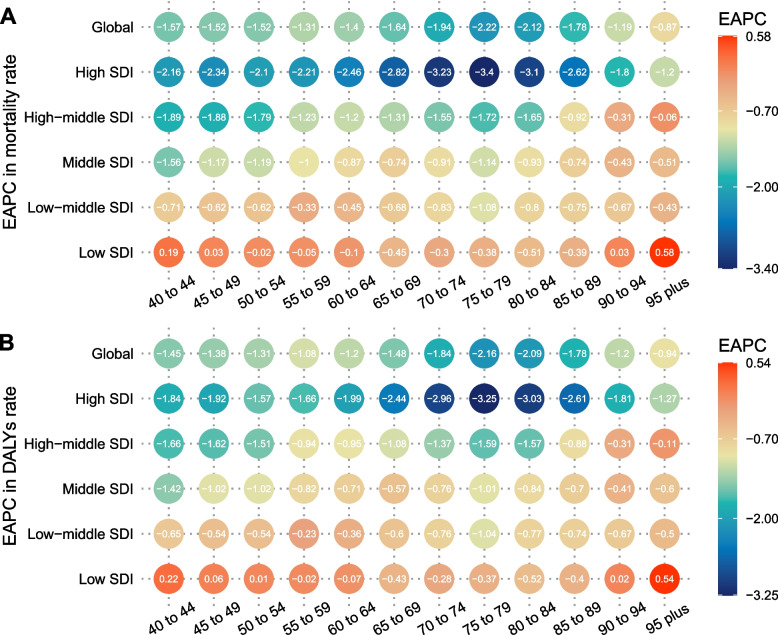
Annual percentage change in mortality (**A**) and DALYs rate (**B**) between 1990 and 2019 by age group and region. Footnote: EAPC, estimated annual percentage change; SDI, sociodemographic index; DALYs, disability-adjusted life-years

Globally, the age-specific mortality rate has decreased among all ages group from 1990 to 2019, with the fastest decrease occurring in 75–79 years old. Separately, the age-specific mortality rates have decreased in low-middle, middle, high-middle, and high SDI regions from 1990 to 2019, among which high-middle and high SDI regions had higher EAPCs in mortality rates in each age group compared to low and low-middle SDI regions. In low SDI region, the age-specific mortality rates have increased in 40–49 years old and increased over 90–95 years old (Fig. 5A). The EAPCs in age-specific DALY rate showed the same pattern as that in age-specific mortality rate (Fig. 5B).

### Factors associated with prostate cancer burden attributable to smoking

**Fig. 6:**
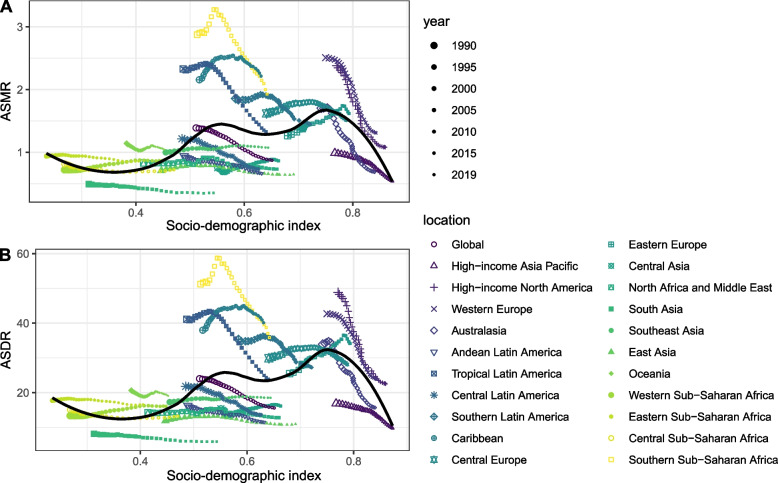
The correlation between smoking attributable prostate cancer in ASMR or ASDR and SDI globally and 21 GBD regions from 1990 to 2019. Footnote: ASMR, age-standardized mortality rate; ASDR, age-standardized DALY rate; GBD, Global Burden of Disease Study

**Fig. 7:**
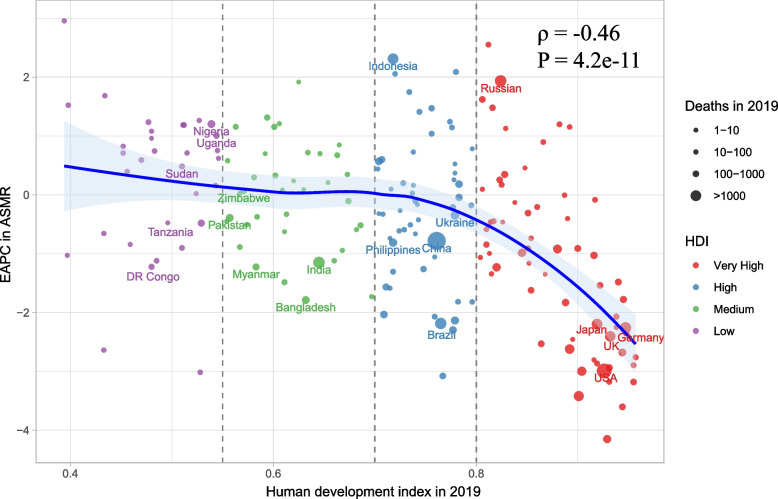
The correlation between EAPC in ASMR and HDI in 2019. Footnote: ASMR, age-standardized mortality rate; HDI, human development index

Overall, the ASMR and ASDR across regions had an M-shaped association with SDI in 2019, with the inflection point around 0.55 and 0.75 (Fig. 6). Moreover, the ASMR and ASDR association with SDI across countries has been visualized. Across countries, as SDI increased, ASMR or ASDR increased until SDI was about 0.75, and then decreased with higher SDI. Based solely on SDI, the ASMR or ASDR was much higher than expected in Zimbabwe, Seychelles, and Dominica (Additional files 6 and 7). The EAPC in ASMR was highly negatively associated with HDI in 2019 (ρ = -0.46, *P* < 0.001), especially with HDI greater than 0.8 (Fig. 7). However, no significant associations were observed between EAPC in ASMR and ASMR in 1990 across different countries (Additional file 7: Fig S6). The same patterns were also observed between EAPC in ASDR and HDI in 2019, and ASDR in 1990 (Additional files 8 and 10).

## Discussion

In this study, we used the latest GBD 2019 data to summarize the epidemiological characteristics of the global burden of smoking-related prostate cancer, and the results showed that 6% deaths and 6.6% DALYs of total prostate cancer among males were attributable to smoking. Globally, the trend of prostate cancer in ASMR and ASDR attributable to smoking slightly decreased over the last 30 years, but the corresponding absolute number of prostate cancer deaths and DALYs has increased by half, which can be partially explained by the aging and growth of the population (Additional file 11). The highest number of deaths occurred in 75–79 years old, and the highest mortality rates occurred in 95 + years old. Furthermore, the spatial distribution and the temporal trend of prostate cancer attributable to smoking were heterogeneous, which showed a complex association with smoking control and prostate-specific antigen (PSA) use.

Tobacco smoke is recognized as a significant risk factor in several genitourinary cancers, likely due to the accumulation of carcinogens in urine. However, in prostate cancer, the link is weak and often overlooked. The biological link between smoking and prostate cancer remains to be elucidated, although several potential mechanisms have been proposed. For example, tissue damage associated with increased exposure to carcinogenic compounds in smoke may cause elevated concentrations of neutrophils, and activate intracellular signaling cascades which in turn stimulate inflammatory gene activation [18]. Alternatively, smoking induces lasting effects on systemic VEGF, which may contribute to systemic hypoxia [19]. Another possibility is DNA methylation. A previous study showed that among males with tumors, smokers had differential methylation profiles across 40 regions in prostate tumor tissue compared to those in nonsmokers, who has a lower risk of recurrence and lethal disease [20]. Finally, an unhealthy lifestyle correlated with smoking such as temper tantrums, poor hygiene, and staying up late also plays a critical role in the risk of developing prostate cancer.

The prostate cancer burden attributable to smoking varied substantially across regions and nations. As our results illustrated, similar patterns of reduction in ASMR and ASDR were observed in High-income North and Tropical Latin America, Western Europe, Australasia, and developed countries of Asia, reflecting widespread adoption of PSA testing and regulatory policy for tobacco smoking. In contrast, rapidly increasing trends in ASMR have been found in Western Sub-Saharan Africa, Central Asia, and Eastern Europe. More specifically, the rise in Western Sub-Saharan Africa is possibly due to an underlying rise in incidence trends combined with a more westernized lifestyle and limited access to treatment [1], whereas Central Asia might be explained by an increased prevalence of risk factors associated with globalization and economic development, such as increased consumption of dietary fat and decreased level of physical activity, and significantly increased smoking tobacco use over the past 30 years (eg, in Afghanistan, Saudi Arabia, Uzbekistan, Lebanon) [4, 21]. The high mortality rates observed in Russia and former Soviet Union countries after implementation of the PSA-based screening could be related to the over-reporting of prostate cancer as an underlying cause of death in death certificates [22]. A similar issue was noted in the USA in 1991 [23].

As was documented, younger male patients with early-onset PCa have risen over the past 3 decades, and had the highest mortality among all age groups, since they were inclined to have a higher risk or metastatic form [24–26]. However, our results showed that the proportion of prostate cancer mortality attributable to smoking in patients younger than 55 years remained stable (Additional file 1: Fig S1). This may be due to lower smoking prevalence and the shorter duration of exposure to smoking in those age groups. Moreover, our research also indicates a slight increase in the proportion of cancer-specific mortality rates attributable to smoking among older adults older than 75 years (Additional file 1: Fig S1). This was probably due to the lag between stopping smoking and developing prostate cancer. The cohort born between 1955–1965 (aged 35–44 years in 2000) was the first cohort to experience a significant decline in smoking prevalence, from 32.0% in 2000 to 21.1% in 2020, while the cohort born before that time remained almost stable over time [27]. Globally, the number of deaths and DALYs, and EAPCs in ASMR and ASDR both showed an inverted U-shaped, with the peak point appearing at 75–79 years old, reflecting peak mortality rates shifted to older age groups (Figs. 4A,B and 5A,B).

Although age, ethnicity and geographic variations are the main risk factors for PCa, socioeconomic status had a modifying role in the effect of smoking on prostate cancer. There was a temporary increase in the burden across countries with lower SDI, peaking among those countries with SDI around 0.75, before a decreasing pattern in countries with higher SDI. The main risk factors for patients with low socioeconomic status are watchful waiting, harsher work environments, higher smoking rates, and less likely to be treated with radical surgery or radiotherapy [28–30], which tend to decline with quality management in healthcare at a higher level of socioeconomic development. Globally, prostate cancer burden attributable to smoking has been declining from 1990 to 2019, but substantially increased in undeveloped countries. Therefore, equity of screening and treatment patterns is expected in these regions.

Three large randomized trials with long follow-ups provide compatible evidence that PSA-screening reduces prostate cancer mortality [31–33]. In the U.S., the age-specific prostate cancer mortality halved over the course of PSA screening and improvements in treatment [34] but the long-term benefits and harms associated with screening remain uncertain since the cost of overtreatment and its side effects such as erectile dysfunction and incontinence [35, 36]. Furthermore, although government action in reducing the prevalence of smoking tobacco has had a major effect, a large implementation gap remains and progress in many countries has slowed in the past 10 years [4, 6]. In the light of the findings, improvement in PSA screening programs, public management policies, and primary tobacco interventions should be tailored based on geographic variation, age, and socioeconomic status. For example, developing countries with elevated mortality rate where PSA-based mass screening has not been widely used still need to introduce nation-wide screening to discover the potentially asymptomatic patients. By contrast, developed countries which have made major progress over the past 30 years were recommended to explore novel biomarkers or radiological imaging with risk-predicting models to avoid harms of screening and promote shared decision making for men aged 55 to 69 years [37].

Previous studies on this topic have explored the relationship between prostate cancer and smoking but did not reveal the heavy disease burden due to smoking and its distribution on a global scale. Our study used the latest GBD data to systematically investigate the smoking-related burden and measure the effectiveness of interventions, but there are still limitations. First, data collection and PSA testing are both imperfect in some underdeveloped countries, which severely affects the reliability and statistical analysis of the data. Second, the enforcement of tobacco control has varied markedly across countries, so is difficult for us to make any association between our findings and changed smoking interventions. Third, there is no data on race and ethnicity in our study, thus we are unable to distinguish from those of socioeconomic status owing to their frequent co-occurrence.

## Conclusion

The burden of smoking-related prostate cancer among males remains a defining challenge in global health, under the background of numerous prostate cancer patients and diminishing potential for further reductions in smoking. Government medical strategy should be emphasized in several high-risk regions, particularly in Eastern Europe, where a steady mortality increase was found. In addition, disease burdens were higher among old people and people in low-SDI countries, posing a long-term challenge to health and economic cost with growth and aging population. Lastly, the findings will give valuable assistance to policymakers in addressing modifiable risk factors and making regulatory changes on smoking-related prostate cancer control and prevention.

## Supplementary Information

**Additional file 1.** 

**Additional file 2:** **Table S1.** Top 10 countries or territories with the highest number of prostate cancer deaths attributable to smoking in 2019. **Table S2.** Top 10 countries or territories with the highest number of prostate cancer DALYs smoking in 2019. **Table S3.** Top 10 countries or territories with the highest prostate cancer ASMR per 10 000  attributable to smoking in 2019. **Table S4.** Top 10 countries or territories with the highest prostate cancer ASDR (per 100,000) attributable to smoking in 2019. **Table S5.** Top 10 countries or territories with the highest or lowest EAPC in ASMR (per 100,000) attributable to smoking, 1990-2019. **Table S6.** Top 10 countries or territories with the highest or lowest EAPC in ASDR attributable to smoking, 1990-2019.

**Additional file 3:** **Table S7.** Percentage change across countries of the fraction of all prostate cancer deaths that are attributable to smoking (95% UI).

**Additional file 4:** **Table S8.** Percentage change across countries of the fraction of all prostate cancer DALYs that are attributable to smoking (95% UI).

**Additional file 5.** 

**Additional file 6.** 

**Additional file 7.** 

**Additional file 8.** 

**Additional file 9.** 

**Additional file 10.** 

**Additional file 11:** **Table S6.** Decomposition analysis of the contribution of population growth and population aging on the global prostate cancer burden due to smoking.

## Data Availability

Data was extracted from an online tool produced by the IHME, which is publicly available called the GHDx (Global Health Data Exchange) query tool (http://ghdx.healthdata.org/gbd-results-tool).
