# Supplementary figures and images for: Global burden of prostate cancer attributable to smoking among males in 204 countries and territories, 1990–2019
Source: BMC Cancer. 2023 Jan 26;23:92. doi: 10.1186/s12885-023-10552-8 (PMC9878877; doi:10.1186/s12885-023-10552-8)

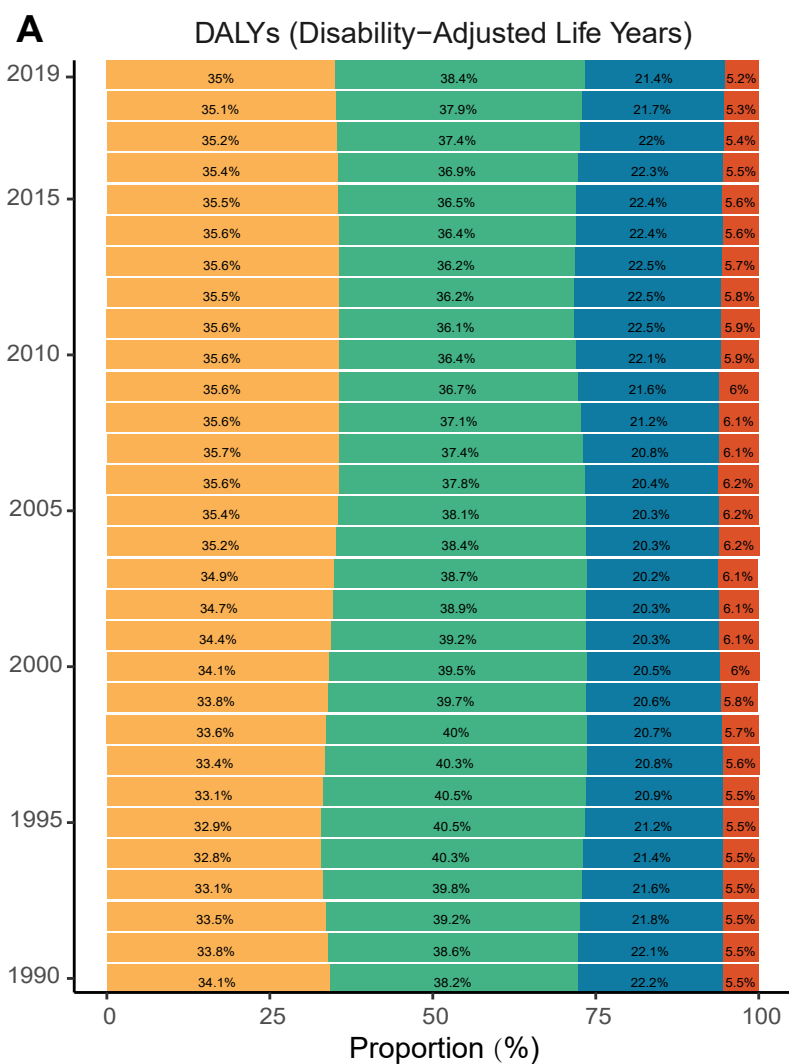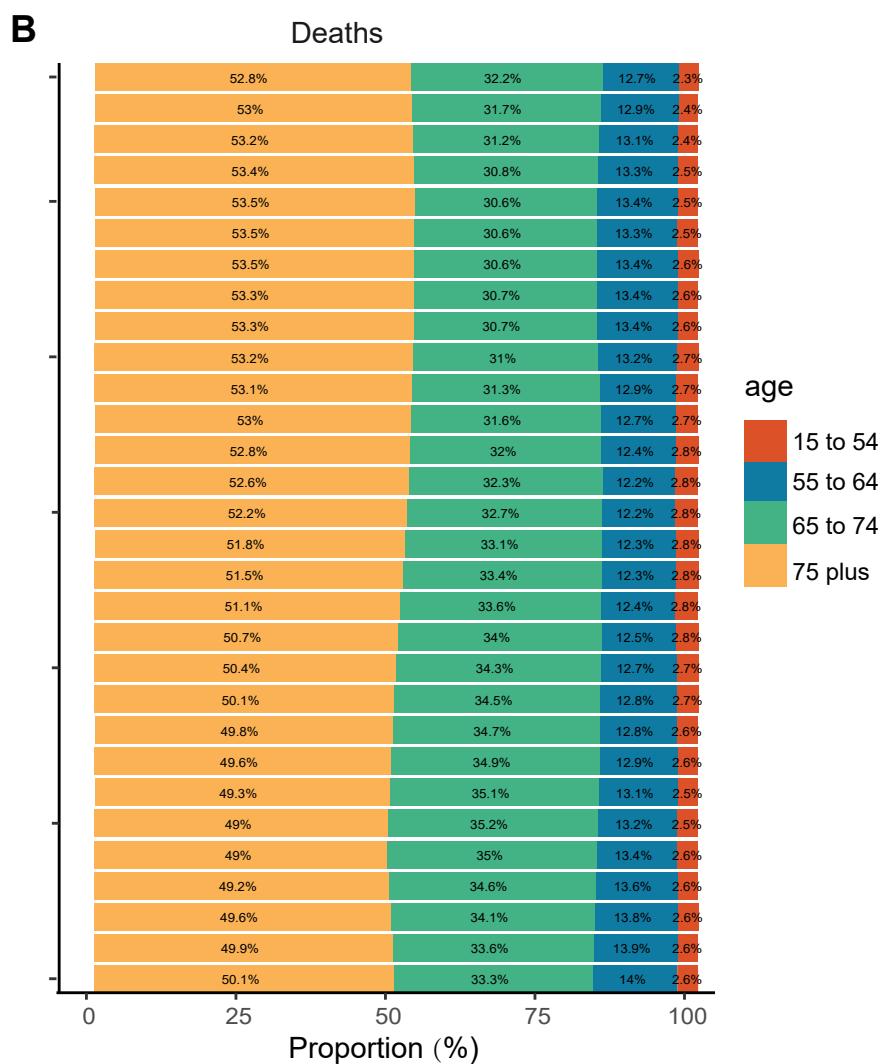

Supplement: Supplementary file 1 — Additional file 1. [file 12885_2023_10552_MOESM1_ESM.pdf]

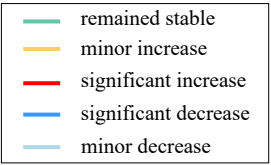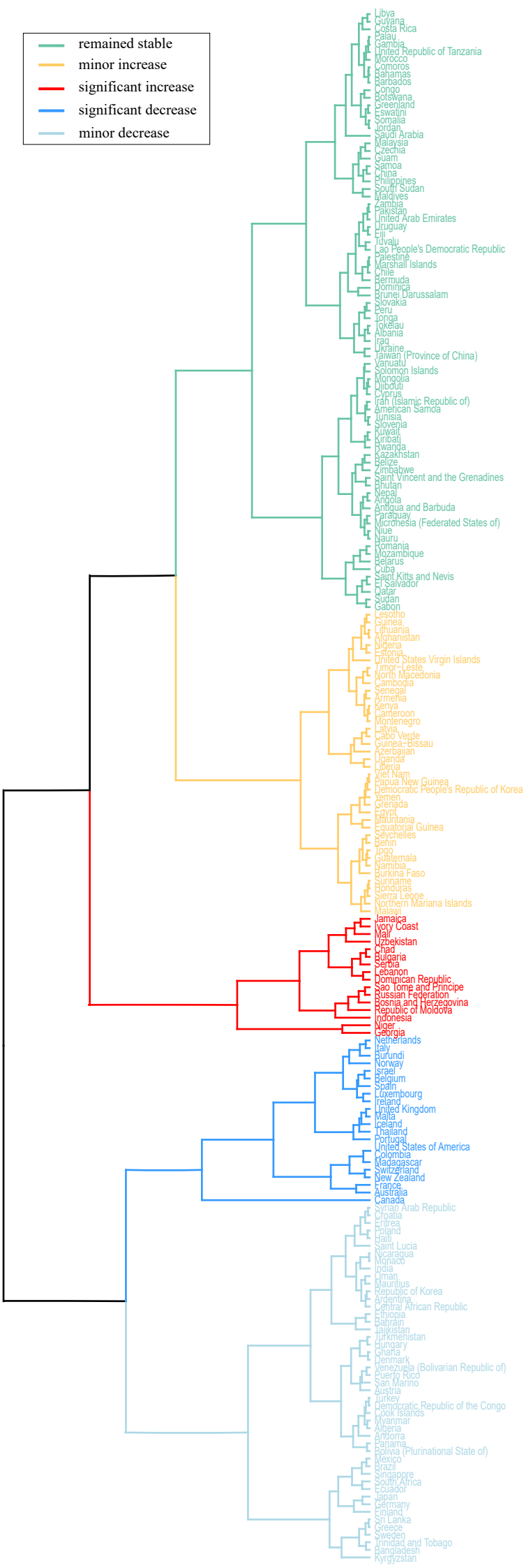

Supplement: Supplementary file 5 — Additional file 5. [file 12885_2023_10552_MOESM5_ESM.pdf]

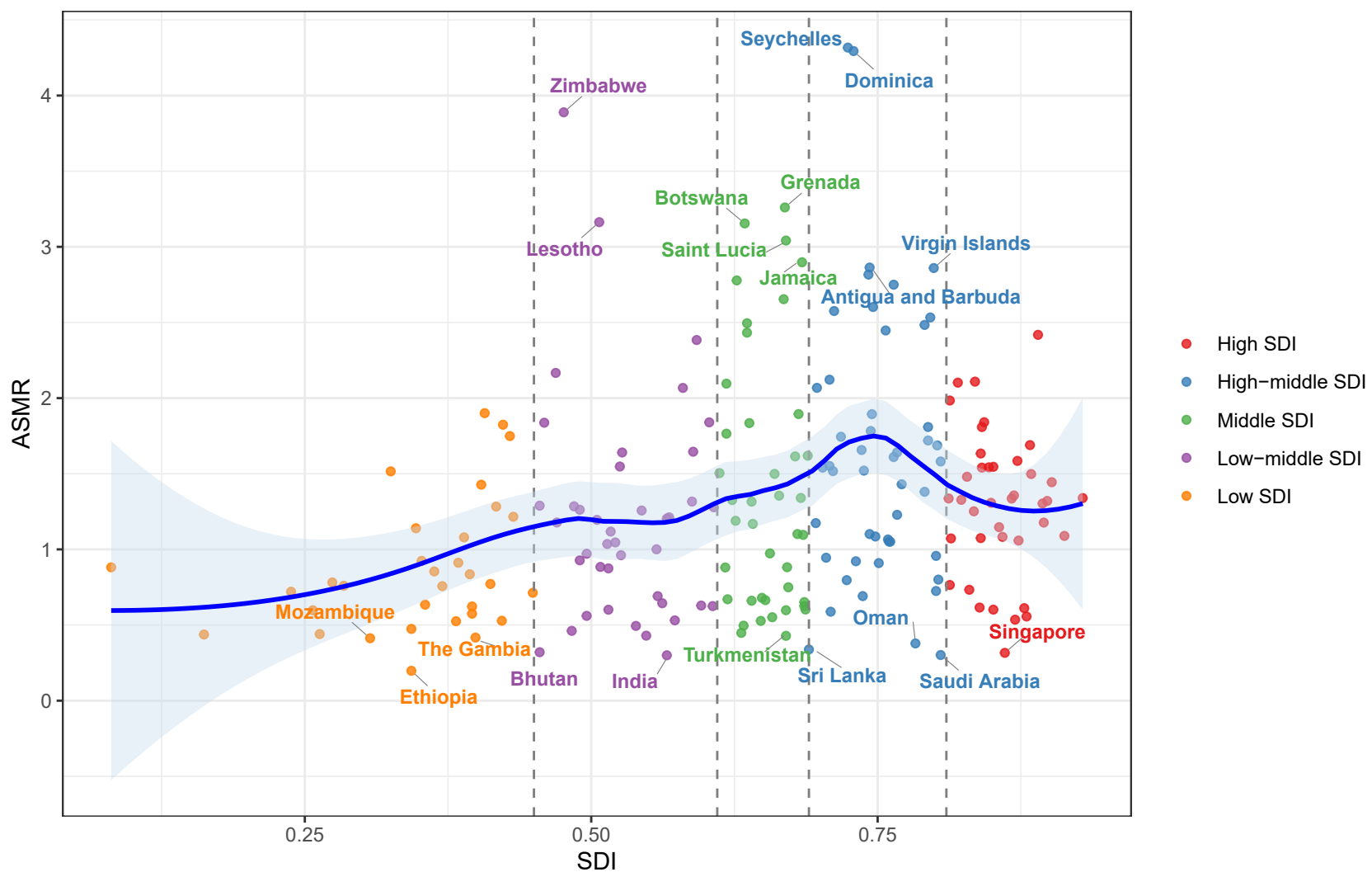

Supplement: Supplementary file 6 — Additional file 6. [file 12885_2023_10552_MOESM6_ESM.pdf]

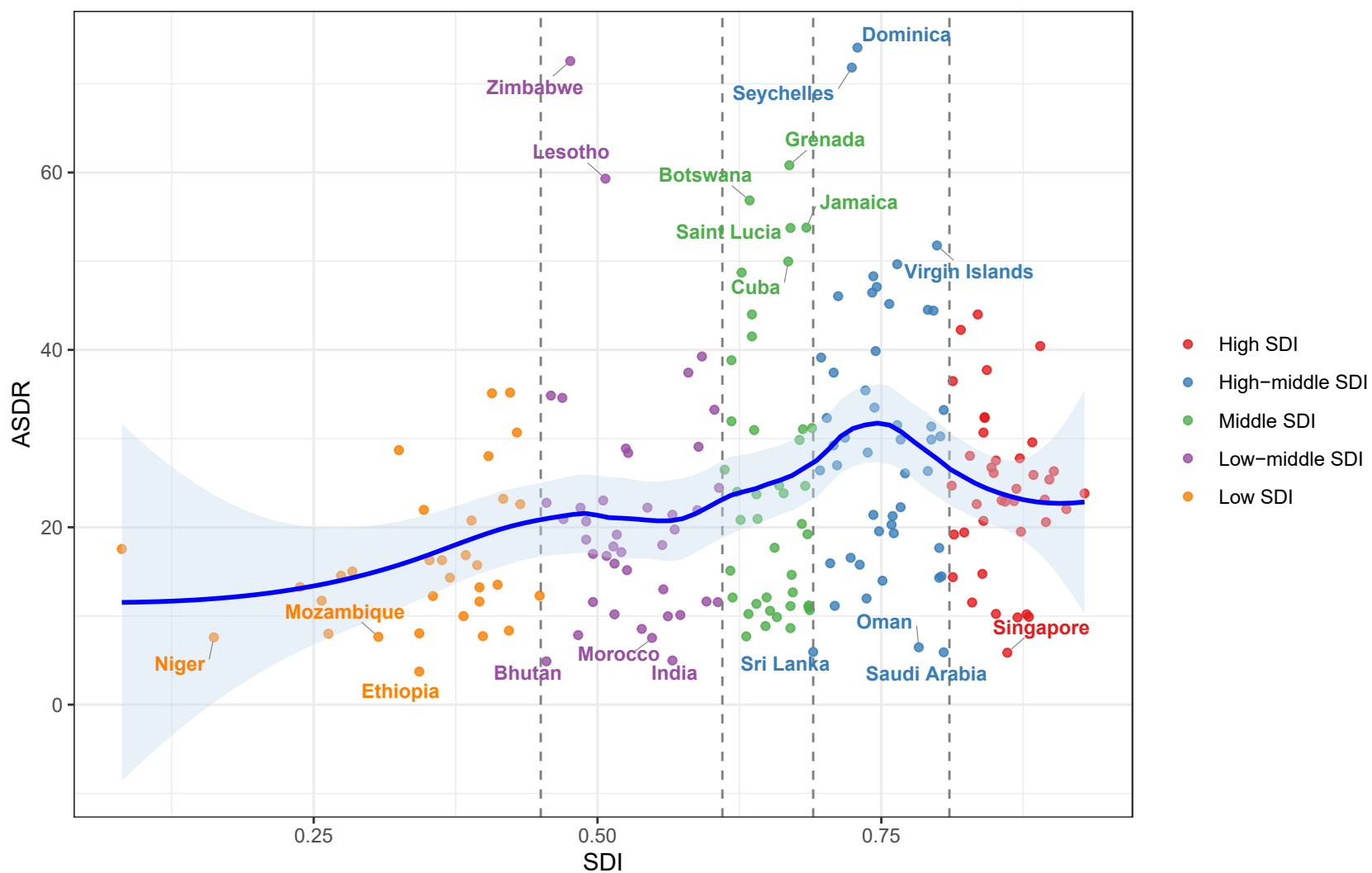

Supplement: Supplementary file 7 — Additional file 7. [file 12885_2023_10552_MOESM7_ESM.pdf]

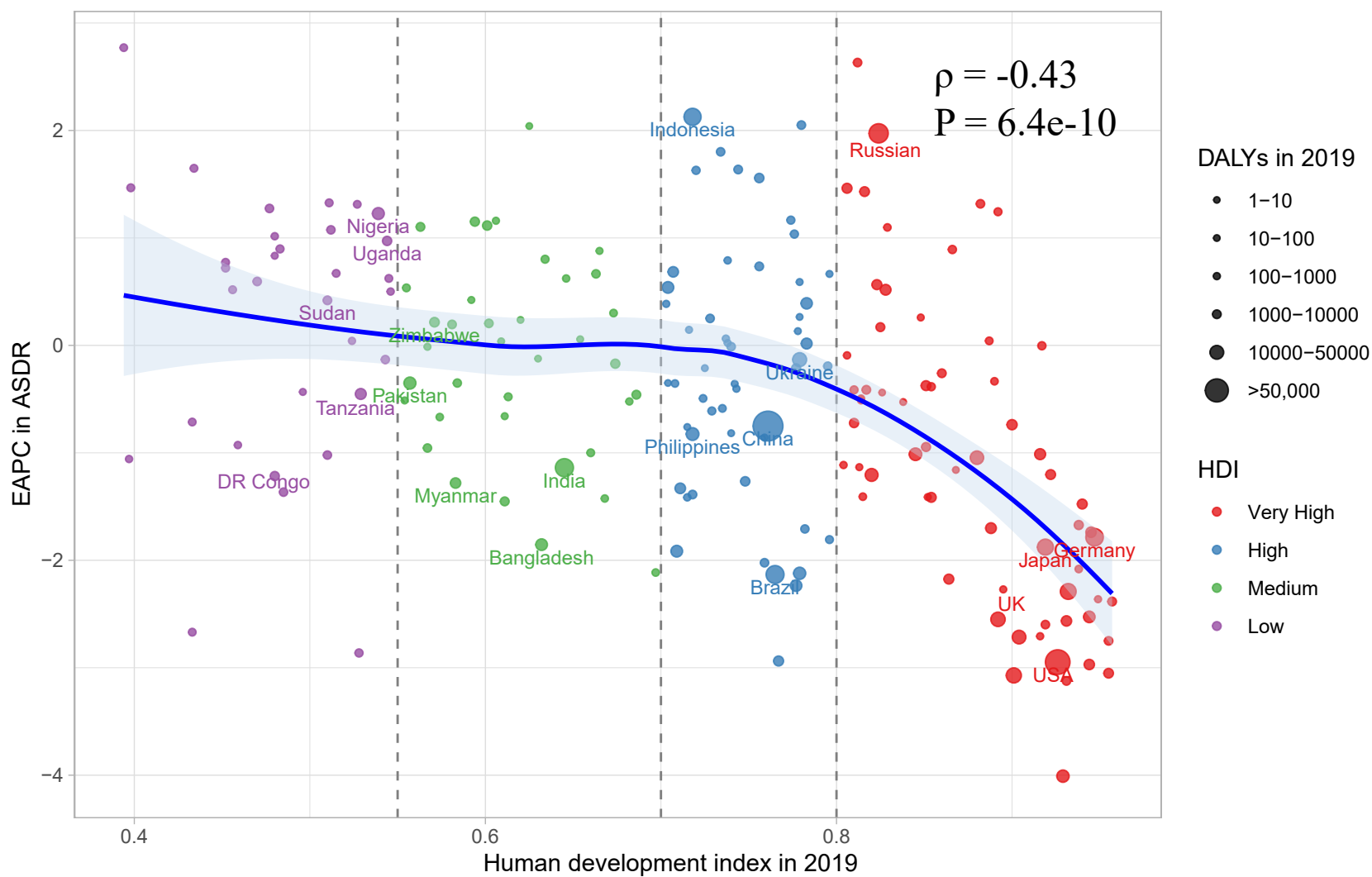

Supplement: Supplementary file 8 — Additional file 8. [file 12885_2023_10552_MOESM8_ESM.pdf]

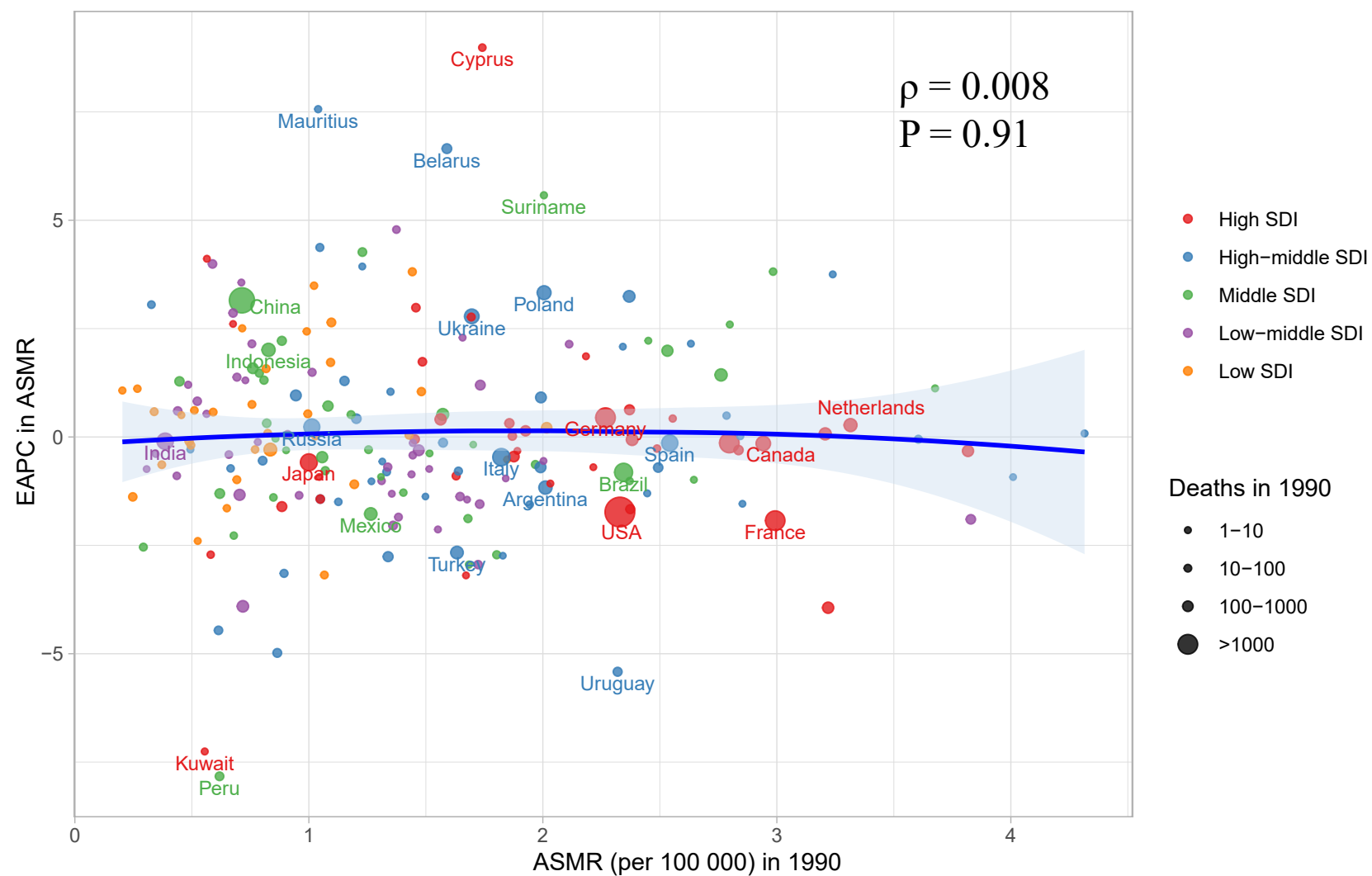

Supplement: Supplementary file 9 — Additional file 9. [file 12885_2023_10552_MOESM9_ESM.pdf]

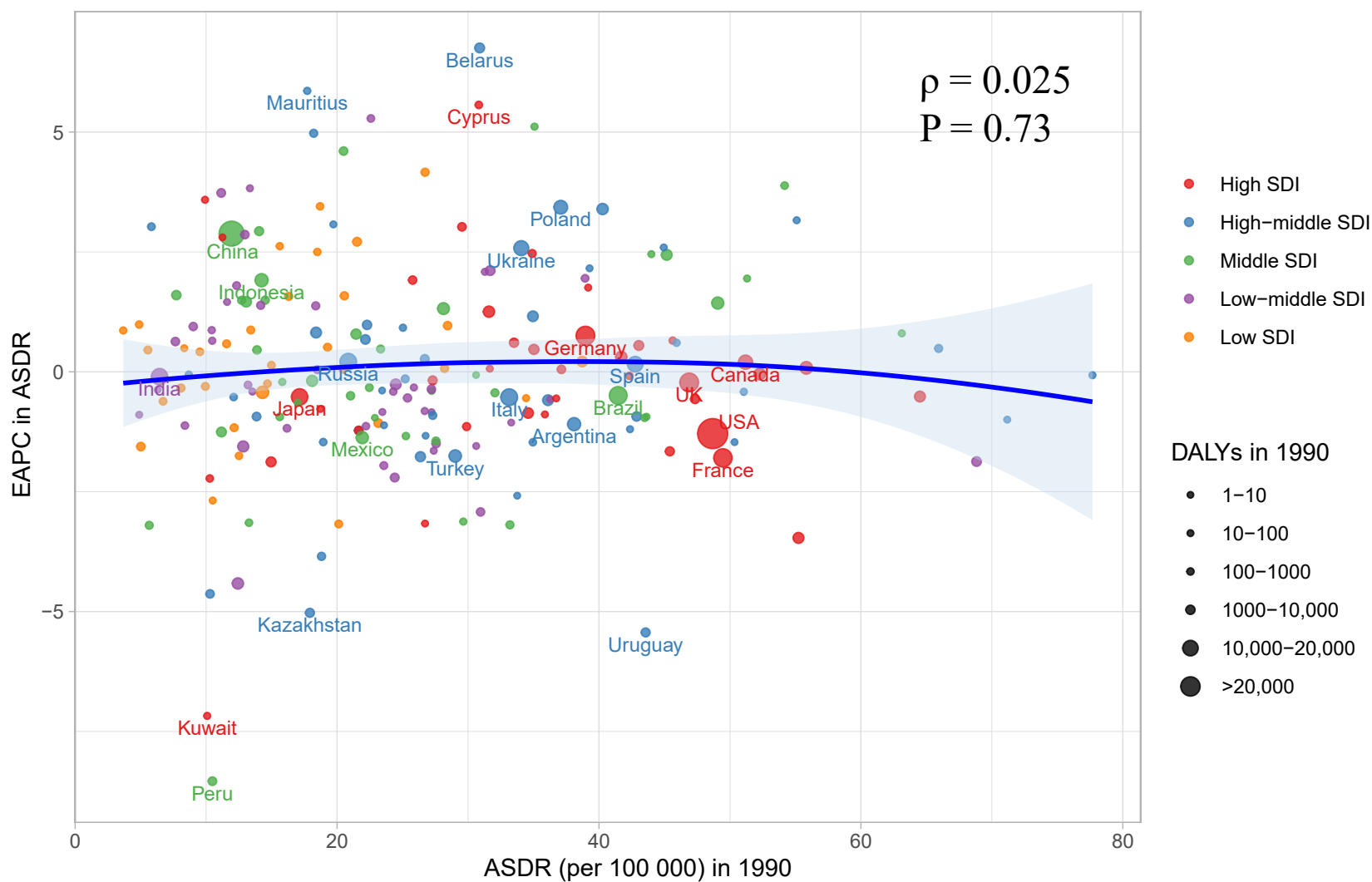

Supplement: Supplementary file 10 — Additional file 10. [file 12885_2023_10552_MOESM10_ESM.pdf]
